# Charge State Dependence of Amino Acid Propensity at Water Surface: Mechanisms Elucidated by Molecular Dynamics Simulations

**DOI:** 10.1021/acs.jpca.0c10963

**Published:** 2021-05-27

**Authors:** Radost Herboth, Geethanjali Gopakumar, Carl Caleman, Malin Wohlert

**Affiliations:** † Department of Materials Science and Engineering, Uppsala University, Box 35, 751 03 Uppsala, Sweden; ‡ Department of Physics and Astronomy, Uppsala University, Box 516, 751 20 Uppsala, Sweden; § Center for Free-Electron Laser Science, DESY, Notkestraße 85, 226 07 Hamburg, Germany

## Abstract

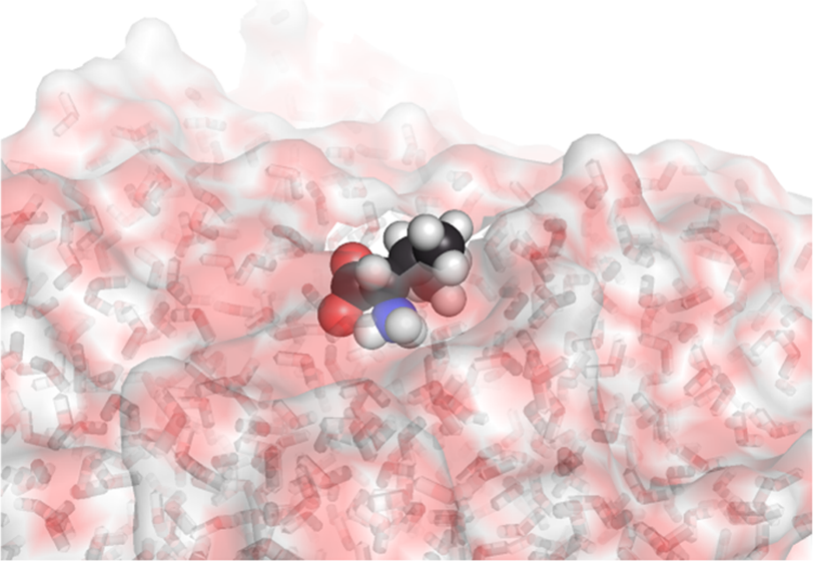

Atmospheric aerosols contain a variety of compounds, among them free amino acids and salt ions. The pH of the aerosol droplets depends on their origin and environment. Consequently, compounds like free amino acids found in the droplets will be at different charge states, since these states to a great extent depend on the surrounding pH condition. In droplets of marine origin, amino acids are believed to drive salt ions to the water surface and a pH-dependent amino acid surface propensity will, therefore, indirectly affect many processes in atmospheric chemistry and physics such as for instance cloud condensation. To understand the surface propensity of glycine, valine, and phenylalanine at acidic, neutral, and basic pH, we used molecular dynamics (MD) simulations to investigate them at three different charge states in water. Their respective surface propensities were obtained by the means of a potential of mean force (PMF) in an umbrella sampling approach. Glycine was found to have no preference for the surface, while both valine and phenylalanine showed high propensities. Among the charge states of the surface-enriched ones, the cation, representing the amino acids at low pH, was found to have the highest affinity. Free energy decomposition revealed that the driving forces depend strongly on the nature of the amino acid and its charge state. In phenylalanine, the main factor was found to be a substantial entropy gain, likely related to the side chain, whereas in valine, hydrogen bonding to the functional groups leads to favorable energies and, in turn, affects the surface propensity. A significant gain in water–water enthalpy was seen for both valine and phenylalanine.

## Introduction

Atmospheric aerosols of micrometer size, on which water condenses and forms cloud droplets, are called cloud condensation nuclei (CCN). One example of CCN are marine aerosol particles formed through bubble bursting induced by wind pressure on the surface of the water.^1,2^ The size distribution of these aerosol droplets influences the optical properties of clouds such as reflectivity and thereby the absorption and reflection of solar radiation.^3^ Activation of a particle to form cloud droplets is explained by the Köhler theory,^4^ which describes the critical supersaturation for the nucleation and growth of droplets using two important parameters: water vapor pressure and surface tension. A decrease in surface tension lowers the critical supersaturation of the water vapor and favors the activation of cloud droplets. As a consequence, smaller-sized cloud droplets are formed, which increase the droplet density and thereby the reflectivity of the clouds.^5,6^ The presence of surface-active organic molecules, for instance free amino acids, in the aerosol is one reason for a decrease in surface tension.^7−11^ Free amino acids are essential components of aerosols^12,13^ and play a significant role in atmospheric chemistry and physics. For example, they can react with atmospheric oxidants,^14,15^ form brown carbon, which absorbs solar radiation,^16^ and contribute to the global nitrogen cycle through atmospheric depositions.^17^ The different amino acids identified in marine aerosol particles are glycine, alanine, valine, proline, serine, methionine, and phenylalanine.^14,17−21^ Some of these dissolved amino acids are surface-active^22^ and affect the hygroscopicity, i.e., the water intake capacity of the aerosols.^23^ Moreover, it has been shown by laboratory experiments that amino acids are potential CCN.^24^ In general, the density of the amino acids at aerosol surfaces influences surface tension and surface reactions, which, in turn, governs the formation of cloud droplets and atmospheric chemistry processes.

Molecular dynamics (MD) simulations of amino acids at the aqueous surface have shown that hydrophilic amino acids like glycine, serine, and alanine prefer to be in the bulk, while amphiphilic and hydrophobic amino acids like valine, methionine, and phenylalanine concentrate at the surface.^25^ An experimental study of aqueous amino acid solutions employing X-ray photoelectron spectroscopy (XPS) confirms this observation.^26^ The amino acids that are less surface-active have less impact on the surface tension and consequently on the nucleation of the cloud droplets.^27^ Simulations of similar systems show that the nucleation dynamics also depend on the curvature of the surface along with the surface density of amino acids.^25^ Additionally, simulations of a mixed solution of glycine and sea salt indicate that the efficiency of the nucleation process is higher in the mixture compared to a solution of only the amino acid.^28^

Interestingly, the pH of the aqueous environment has not been addressed in any of these experimental or simulation studies. However, since marine aerosol particles are subjected to different chemical reactions and radiation, the chemical composition inside these particles will not be constant. For example, they pick up nitric and sulfuric acids as they age in the atmosphere^29,30^ implying a change of pH in aerosol over time. Marine aerosols arising from slightly basic sea were also observed to be acidic. Furthermore, there are results indicating the presence of a pH gradient inside the aerosol particles.^31^ Organic molecules in the aerosol, such as amino acids, show a pH-dependent protonation of the functional group(s) and will thereby be in different charge states depending on the pH of the environment. Whether the amino acid charge state has an influence on their surface propensity or not, and if so in what sense, has to our knowledge not been reported before.

The present study, therefore, aims to fill this gap by employing MD simulations to investigate the surface affinity of three amino acids, glycine (GLY), valine (VAL), and phenylalanine (PHE), in aqueous solutions at different charge states, as representative systems of varying pH environment.

## Methods

Surface propensity was obtained from the potential of mean force (PMF) calculated from MD simulations as a function of distance from the water surface for three selected amino acids; GLY, VAL, and PHE. For each amino acid three different charge states were investigated as models of representing different pH. The charge states were cationic (low pH), zwitterionic (neutral pH), and anionic (high pH). The PMF was obtained from umbrella sampling (US)^32^ with starting configurations generated from a steered MD (COM pulling) simulation. A slab of 2165 water molecules with a volume of 4 × 4 × 4 nm^3^ was placed in the center of a box that extended to 12 nm in *z*-direction, creating a water/vacuum interface.

The procedure was analogous to the one used in previous simulations of organic molecules at the water surface.^33^ Each system was first equilibrated, and subsequently the pulling simulation was run along the *z*-direction, with the reaction coordinate *r* being the center of mass distance between the water slab and the amino acid. A spacing of 0.1 nm was used for the selection of configurations for the umbrella simulations. Each simulation was equilibrated 100 ps and subsequently run 100 ns using a stochastic dynamics integrator, with the amino acid being restrained by a harmonic potential. The PMF was calculated from the simulations using the weighted histogram analysis method (WHAM) in the GROMACS command gmx wham(34) with 41 bins, while setting the free energy of the initial point (*r* = 1.0 nm) to zero. All simulations were performed using the GROMACS 2018.6 software package.^35^ A detailed description of all simulation parameters is presented in the Supporting Information.

The PMF corresponds to the free energy along a reaction coordinate (*r*) relative to a reference state, which in this case was the free energy at 1 nm, i.e., the free energy of a system with the amino acid in bulk water. Hence, the difference between two states along the PMF curve is considered a difference in Gibbs free energy, Δ*G*, which may be further decomposed into enthalpy (Δ*H*) and entropy (−*T*Δ*S*) contributions
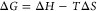
1The enthalpy Δ*H* can be obtained directly from MD as the difference in time-averaged potential energy between a certain umbrella simulation and a reference state. The entropy term −*T*Δ*S* is then given by eq 1 by subtracting the enthalpy from the PMF. Of primary interest in this study is the free energy of adsorption, i.e., the difference in free energy for the system having the amino acid residing at the surface compared to a reference state with the amino acid in the bulk.

It has been suggested from interfacial thermodynamics calculations by Ben-Amotz^36^ that only the solvent–solute interactions can contribute to the entropy term, since the solvent–solvent contribution to the entropy is canceled out by the solvent–solvent contribution to the enthalpy. However, in this study, such a decomposition would not impact our results nor our conclusions. To relate our present work to earlier studies of the PMF of molecules at water surfaces, we have, therefore, chosen to use the free energy decomposition
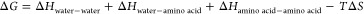
to gain qualitative insight into surface preference based upon the discussion in a previous study by Hub et al.^33^

The force fields employed here are classical, hence, they are neglecting the electronic polarization at the surface, which indeed has an influence on the interfacial behavior of the amino acids. For instance, a published study on the topic found that polarizability contributions represent around one-third of the total positive contributions to water surface tension.^37^ In concordance to that, surface tension of SPC/E water has previously been found to be underestimated, resulting in a value of 61.3 mN·m^–1^ compared to the value of 68.65 mN·m^–1^ using a polarizable force field.^38^ Both models underestimate the experimental value of 71.972 mN·m^–1^. In addition, polarization of the two amino acids containing hydrophobic tails at the interface would also affect the results since polarizable force fields generally yield a better description of the hydrophobic effect.^39^ With this in mind, our choice might at a first glance not seem optimal. However, we believe that for this study, classical force fields are the better option. In the following, we will motivate this choice.

Classical nonpolarizable force fields have successfully been used to model experimentally observed behavior of organic molecules on water surfaces in multiple earlier studies.^40−44^ Specifically, in Walz et al.,^41^ Werner et al.,^42^ and Ekholm et al.,^43^ we have used XPS to measure the structure of organic molecules on water surfaces and compared the experimental observations to MD simulations using classical force fields. In those studies, the observations from simulations and experiments were in agreement, and we were able to use the simulations to better interpret the XPS spectra. The studies included amphiphiles,^41^ carboxylic acids,^42^ and alkyl amines.^43^ Given the structural similarity of these molecules compared to the amino acids simulated here, we expect classical force fields to be able to mimic the surface behavior well enough to draw the conclusions we do.

In contrast to the classical force fields we employ here, the polarizable force fields available for the systems that we are simulating are not well tested for our property of interest, surface propensity. In addition, although polarizable models are a better choice when the density of water is close to the gas state, their performance is far from satisfactory in condensed phases.^45^ It has even been concluded that their reparametrized nonpolarizable counterparts predict many properties of water with greater accuracy.^45^ We have therefore employed classical force fields that we know are not perfect but, on the other hand, are well documented.

We thus used the OPLS-AA force field^46^ for the amino acids and the SPC/E water model^47^ for the majority of our simulations. The combination of OPLS-AA and SPC/E has been found to perform excellently in reproducing experimental hydration enthalpies and entropies, as well as showing good performance for the calculation of solvation-free energies.^48^

However, to evaluate force field dependent effects, we also simulated a subset of the systems using the generalized Amber force field (GAFF)^49^ with the TIP3P^50^ water model. The GAFF parameters for the anionic and cationic states were created as described in Caleman et al.,^51^ and the zwitterion was created based on the parameters in the anion and the cation. The simulations using the two different classical force fields (GAFF and OPLS-AA) agree to the extent that the conclusions we draw in this study do not seem to be dependent on whether we use one or the other.

A more practical reason to use classical force fields is that the PMF simulations are computationally demanding. Nine different systems with an amino acid dissolved in 2165 water molecules, each of them simulated 100 ns in 41 different umbrella windows along the reaction coordinate, is simply not feasible with a polarizable force field.

Overall, we hypothesize that the neglection of electronic polarization will have a small impact on the estimations of surface propensity of amino acids within the present study. To find out exactly how the results would be affected needs further investigation, possibly with surface-sensitive experimental techniques such as XPS, and is out of the scope of this work. The reader must therefore bear in mind that, as in any MD study, the results presented here rely on the accuracy of the underlying force fields, and their ability to sample phase space of the amino acids in both bulk water and in the vicinity of a water/vacuum surface. For amino acids in general, the hydration free energy of hydrophobic side chains is one of the main driving forces behind the affinity of amphiphilic peptides to the water interface.^52^ Therefore, the choice of OPLS-AA and SPC/E water is reasonable since this combination has shown previously to perform well on this particular property.

## Results and Discussion

**Figure 1:**
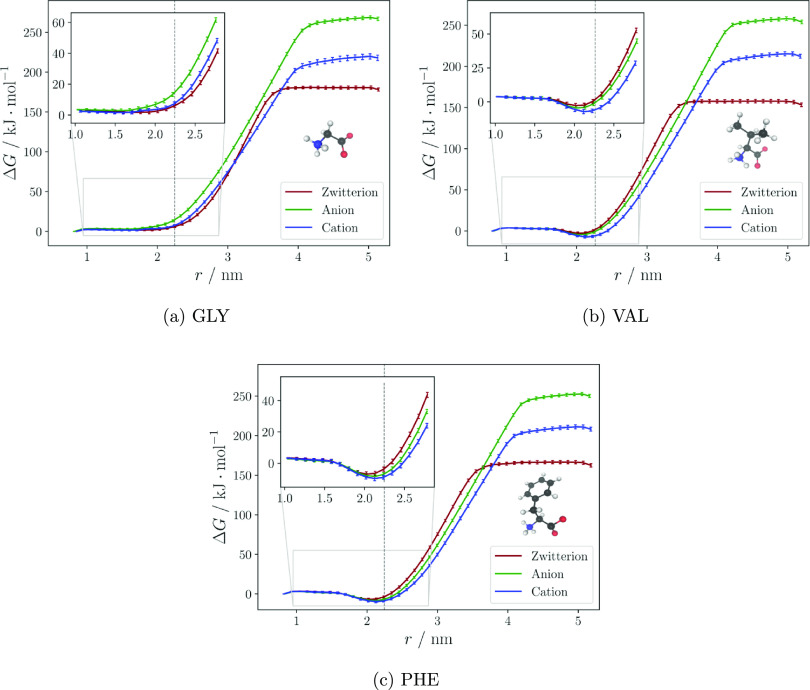
Free energy Δ*G* or potential of mean force (PMF) over the reaction coordinate *r* for the three amino acids, where *r* is defined as the distance between the amino acid and the water slab’s respective centers of masses. The PMF is calculated relative to the bulk, i.e., all values are relative to the first point at *r* = 1.0 nm. The dashed gray line indicates the Gibbs dividing surface.

The free energy of all three amino acids in each charge state as a function of *r*, the distance between the amino acid and the center of the water slab, is shown in Figure 1. The interphase region (1.6 nm ≤ *r* ≤ 2.8 nm) is centered at Gibbs dividing surface, which corresponds approximately to the point at which water density is half of its bulk value (the exact definition and procedure can be found under “Further Methods” in the Supporting Information). Surface propensity corresponds to a minimum in the interphase region and is not seen for any of the GLY ions but on the contrary for all VAL and PHE charge states.

The larger molecules of VAL and PHE show distinct minima, with the cation minima being the deepest, located just below the Gibbs dividing surface between *r* = 2.0 and 2.1 nm (insets in Figure 1b,c). The VAL zwitterion and anion behave similarly, whereas the PHE anion curve is closer to its cation minimum than its zwitterion. When comparing the cation minima for both amino acids, the difference in surface propensity between VAL and PHE is 2.6 kJ·mol^–1^ with the PHE cation being the deepest. Although no experimental data comparing amino acid surface propensity at different charge states was found, the results of the zwitterions are in concordance with previous calculations of free energy of transfer from solution to surface from surface tension measurements of amino acid solutions,^22^ also indicating higher propensity for PHE compared to VAL around their respective isoelectric points. The same study showed, like our simulations, no surface propensity of GLY (cf. Figure S9).

### Free Energy Decomposition

**Figure 2:**
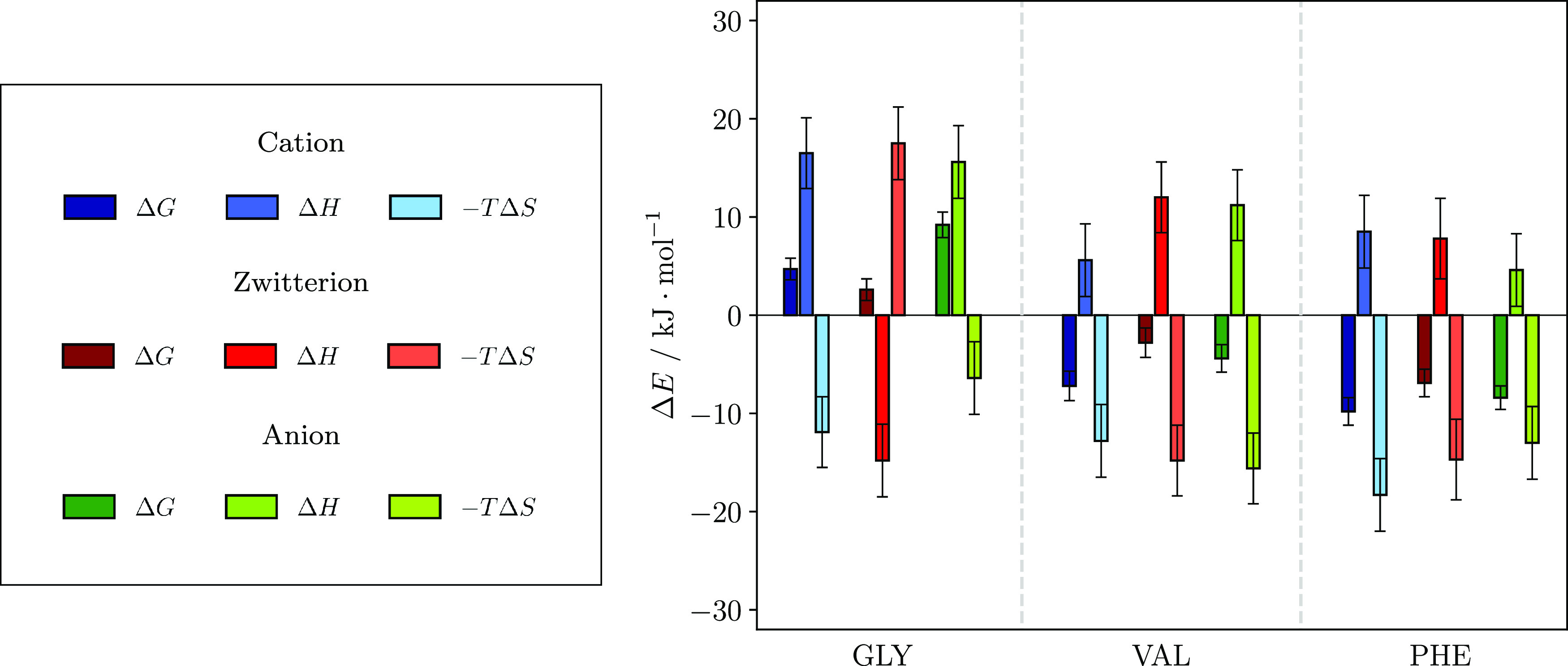
Energy decomposition at the interface according to eq 1. Each color group denotes one charge state (see legend on the left). The term Δ*G* is the difference of free energy at the PMF minimum to the free energy in bulk (*r* = 1.0 nm) and the sum of the other contributions; Δ*H* is the same difference, but in enthalpy contribution, and −*T*Δ*S* is the difference in entropy contribution between the same points. The left bar in each color group then denotes Δ*G* (dark blue, dark red, etc.), which can be split up in two contributions: The middle bar, Δ*H* (blue, red etc.), and the bar on the right, the entropy contribution −*T*Δ*S* (light blue, pale red etc.).

To understand the mechanisms causing attraction of VAL and PHE to the surface, the very subtle balance between enthalpy and entropy contributions to the free energy of adsorption was considered. The difference of the terms in eq 1 at each PMF minimum and at their corresponding reference state at *r* = 1.0 nm were therefore investigated. Figure 2 shows Δ*G* (left bar, dark colors) together with its contributions from enthalpy Δ*H* (middle bar) and the entropy product −*T*Δ*S* (right bar) at the minima.^a^

Almost all Δ*G* values consist of a positive enthalpy and a negative entropy contribution, hence a general gain in entropy and an enthalpy penalty for the amino acids upon transition from bulk to surface, when taking all aspects of interactions into account.

The free energy decomposition of GLY will be briefly discussed here, even though it does not suggest any surface propensity. For GLY cation and anion, the enthalpy penalty outweighs the entropy gain, hampering the transfer of the amino acid to the surface. Surprisingly, the zwitterionic state shows another mechanism. Here, the entropy change is positive, while the enthalpy becomes more advantageous at the surface, although the contributions still sum up to a slightly positive Δ*G*. One possible explanation is that, in a zwitterion, both functional groups carry a charge and therefore the ion interacts strongly with the surrounding water molecules, both in bulk and at the surface. This will be discussed later in the context of water–water interactions in the presence of the amino acids. The origin of the positive entropy difference is less clear. We speculate that its high charge concentration and fairly small size could lead to unfavorable arrangements of water molecules at the surface and subsequently to entropy decrease. It could be interesting to note that at lower temperature the entropy contribution would decrease and possibly give rise to surface affinity for GLY zwitterions.

The mechanisms behind the surface affinity of VAL and PHE will be more extensively discussed, specifically from two aspects: the structural differences between the amino acids on the one hand, and the differences between the charge states, on the other hand. The former pertains to the side chain, while the latter is due to differences in the amine and carboxyl moieties.

As seen in Figure 2, the PHE cation has a pronounced entropy contribution giving rise to its deep minimum, while the VAL cation surface propensity is enhanced because it has a significantly smaller enthalpic penalty at the surface than its other charge states, causing the minimum to be the deepest. Both cases lead to a distinct Δ*G* minimum at the surface. Considering the contributions not only at the minimum but also in the vicinity of the minimum, it was found that VAL cations are interacting advantageously with water while approaching the surface (cf. Figure S2). This was not seen for the other two charge states of VAL or any ion of PHE, which were dominated by entropy gain throughout the entire region of the minimum.

**Figure 3:**
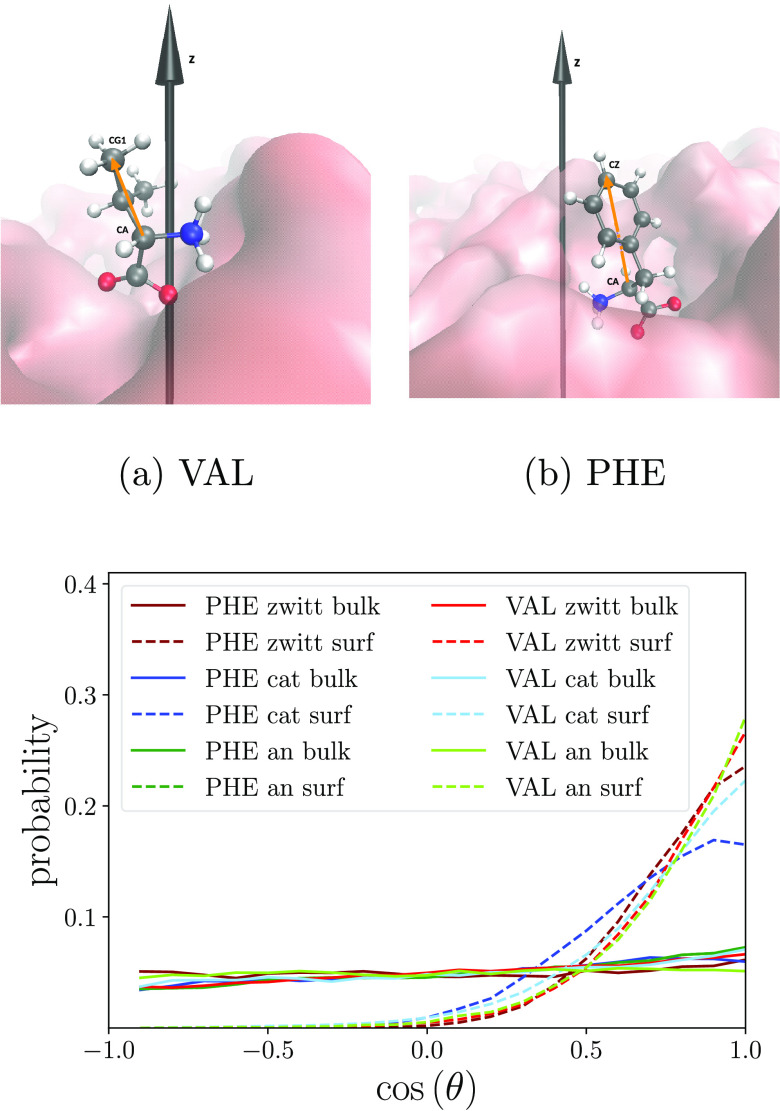
Top: VAL and PHE zwitterions with z-axis (black arrow) and CA-CG1 and CA-CZ vector (yellow arrow). Bottom: Probability distribution of the cosine of the angle θ between the *z*-axis and the CA-CZ vector (PHE) or the CA-CG1 vector (VAL). cos (θ) = 1 means that the hydrophobic part of each amino acid is pointing out from the surface. Bulk data is obtained from *r* = 1.0 nm and surface data from *r* = 2.0 nm (cat: cation, zwitt: zwitterion, an: anion).

It is not possible to pinpoint the reason for this favorable entropy from the results shown here, and both amino acid and water entropy will change. The amino acid has several degrees of freedom, one originating from its total rotational orientation. Its dependence on location with respect to the water surface is further illustrated in Figure 3. Here, the orientation is characterized by a parameter defined as cos (θ), where θ is the angle between the surface normal direction (*z*) and a distance vector between Cα and the end of the respective hydrophobic side chain. In bulk, all molecules show a random orientation distribution with no directional preference, whereas at the surface, an orientation with the hydrophobic part pointing outward is clearly preferred. Consequently, the entropy gain cannot be an effect of increased rotational freedom for the amino acids at the surface. However, the surface cations (especially PHE cations) show a relatively broad distribution, indicating more rotational freedom compared to the other two charge states and hence a comparatively smaller entropy penalty. The determining reason for the overall entropy gain remains unclear, although it is most likely due to water entropy in combination with other entropy terms (translational, vibrational, etc.) of the amino acid.

Water entropy will be affected by the change of location of the amino acid, in that there will be an entropy gain upon filling the cavity that was occupied by the amino acid with water. Simultaneously, there will be a entropy penalty at the water/vacuum surface, as some of the water molecules have to go into bulk where their mobility is decreased due to an increased number of hydrogen bonds per molecule.^53^ These two contributions will thus compete. Moreover, they are size-dependent, as smaller molecules perturb the hydrogen bonding network only slightly and the structure of the solvent is mostly retained.^54^ We do only consider the total entropy, not the water entropy separately and it is, therefore, impossible to say which effect dominates at this point. It may be concluded, though, that all systems except the GLY zwitterion show more favorable total entropy when the amino acid is at the surface.

### Enthalpy Decomposition

**Figure 4:**
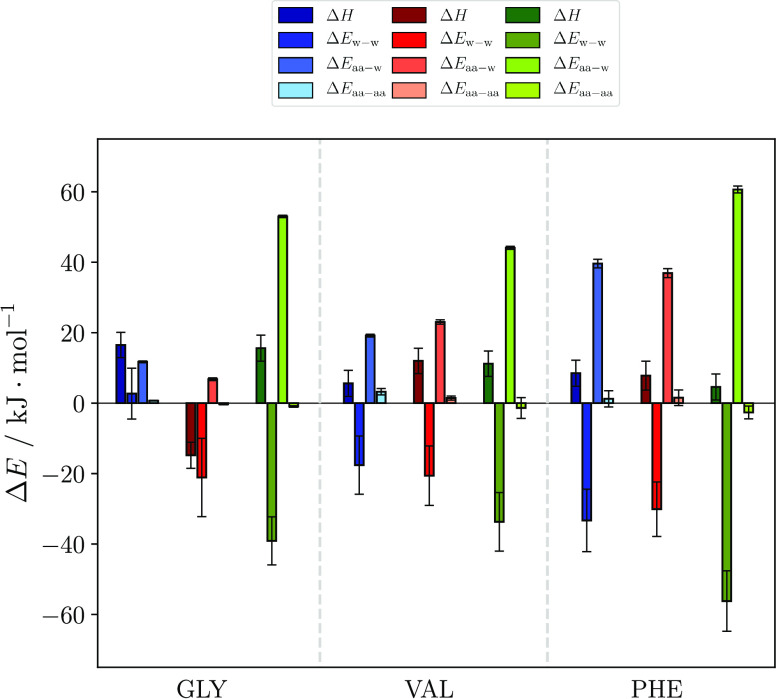
Enthalpy (Δ*H*) decomposition into relative interaction energies between water molecules (Δ*E*_w–w_), amino acid and water (Δ*E*_aa–w_), and amino acid self-interaction (Δ*E*_aa–aa_) at the surface. Each color group denotes one charge state: blue for the cationic state, red for the zwitterionic state, and green for the anionic state; all energies are given as the difference between PMF minimum and the first point of the PMF (*r* = 1.0 nm).

The enthalpy contribution (Δ*H*), limiting the transfer of amino acids to the surface in all cases except for GLY zwitterions, is in contrast to the entropy obtained directly from MD and may be analyzed in terms of individual groups interacting with each other. Figure 4 shows the decomposition of Δ*H* (left bars, dark colors) into the contributions from pairwise Coulomb and Lennard-Jones interactions between water molecules (Δ*E*_w–w_), water and amino acid (Δ*E*_aa–w_), and amino acid self-interaction (Δ*E*_aa–aa_).

Overall, a balance between the gain in water–water and the loss in amino acid–water interactions dominates the picture. The intramolecular interaction of the amino acid with itself contributes much less in comparison, although some small contributions can be seen in the VAL cation and in the PHE anion. In the following sections, these contributions and their mechanisms are discussed in more detail.

#### Water–Water Interaction

To provide a full picture of the contributions to the potential energy of the systems, the difference in water–water interaction upon transition of the amino acids from bulk to surface cannot be neglected, as shown in Figure 4. The key feature here is an enthalpy gain for water–water interactions, especially for VAL and PHE, when moving the amino acid from bulk to surface. These contributions are most significant in the VAL anion (to a lesser degree also in the GLY anion) and all charge states of PHE but always compensated for by a penalty in the amino acid–water interaction.

As mentioned before, this is not seen for the GLY zwitterion, where the water–water interaction energy instead leads to an effective negative enthalpy. Since in this molecule both functional groups carry a charge, they will have a strong influence on the surrounding water molecules. Indeed, water–water interactions in the presence of this ion show a favorable contribution that is not counteracted by an enthalpy penalty from the interaction of amino acid and water (cf. Δ*E*_aa–w_ in Figure 4), which adds to the stabilization through enthalpy. As pointed out earlier, the entropy of this ion instead counteracts surface affinity.

#### Amino Acid–Water Interaction

**Figure 5:**
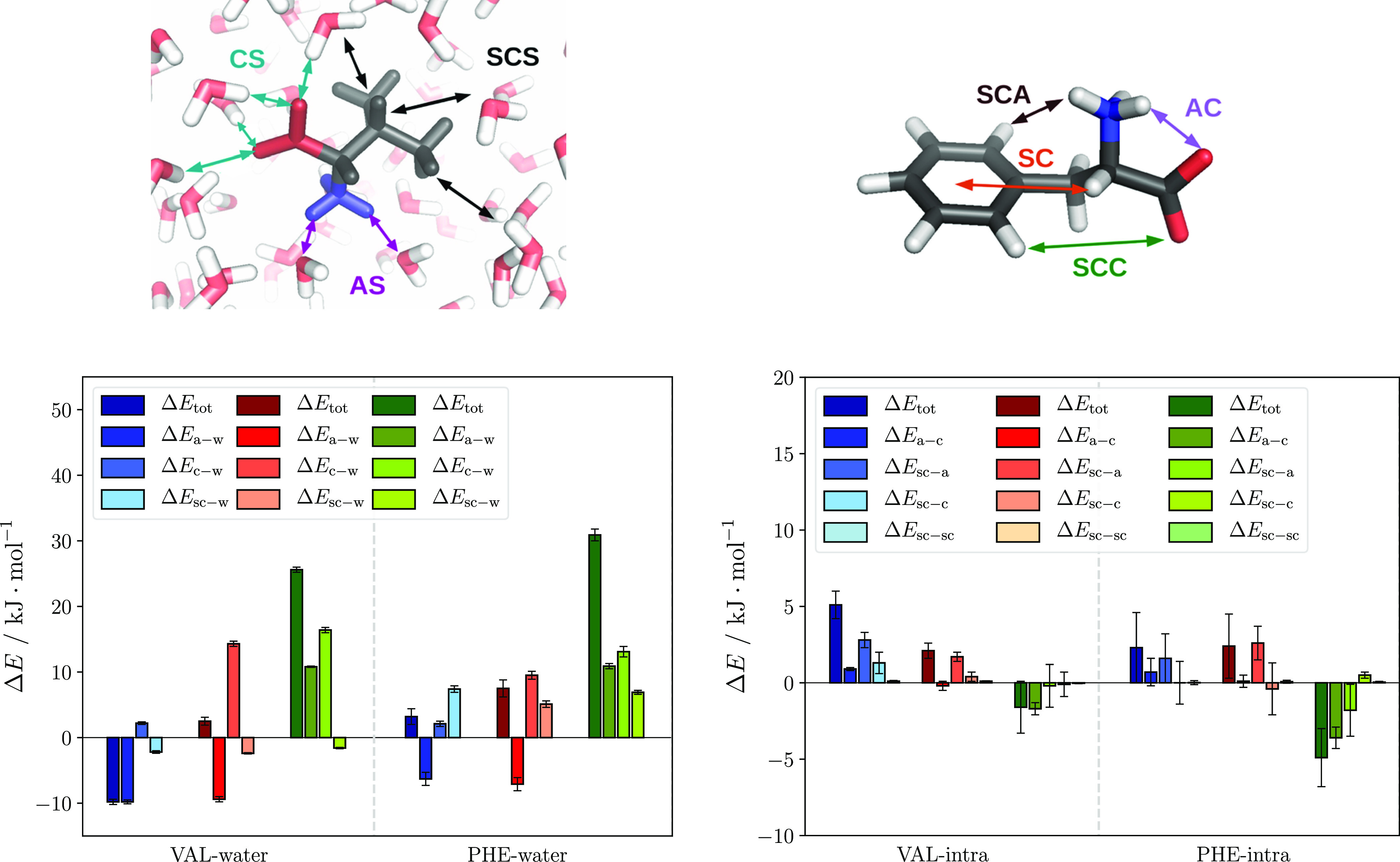
Short-ranged Coulomb interaction energy differences between surface and bulk (Δ*E*_tot_) for amino acid–water interaction (left) and amino acid self-interaction (right, includes intramolecular 1–4 interactions). The total interaction energy in both is broken down into contributions from individual groups, which are illustrated above the respective graphs as the sum of short-ranged (and also the 1–4 intramolecular) interactions from amino acid–water (left) and intramolecular amino acid (right). Each total energy Δ*E*_tot_ is separated into contributions from different groups. Illustrations of these contributions are provided above both graphs (left: VAL, right: PHE). Color coding: each color group denotes one charge state: blue for the cationic state, red for the zwitterionic, and green for the anionic state. Abbreviations: total energy (tot), amine group (a), carboxyl group (c), side chain (sc), and water (w). Notes: The carboxyl group is present as the protonated form of COOH in the cation and deprotonated as COO^–^ in anion and zwitterion, whereas the amine group occurs as NH_2_ in the anion and in the protonated form NH_3_^+^ in cation and zwitterion. All energies are given as the difference between PMF minimum and the first point of the PMF (*r* = 1.0 nm). For clarity, the bottom panels do not scale the same way.

Next, the equally important interactions between water and amino acids will be discussed. For clarity, only VAL and PHE are shown in Figure 5 due to the lack of surface propensity in GLY. Moreover, in this figure, only the short-ranged Coulomb term is considered since it contains the major changes in the amino acid–water interaction upon transition to the surface. Both GLY results and the absolute short-ranged interaction energies can be found in the Supporting Information (Figures S3 and S4).

The contribution from interaction between amino acids and surrounding water molecules is qualitatively different for different ions as seen in the total intermolecular Coulomb energy Δ*E*_tot_ in the left panel of Figure 5. For the VAL cation, amino acid–water interactions are more favorable close to the surface than in bulk, in contrast to the PHE cation, which has a small but significant enthalpic penalty from these interactions. Moreover, the anionic and zwitterionic states always show such a penalty in Δ*E*_tot_, independent of the type of amino acid.

**Figure 6:**
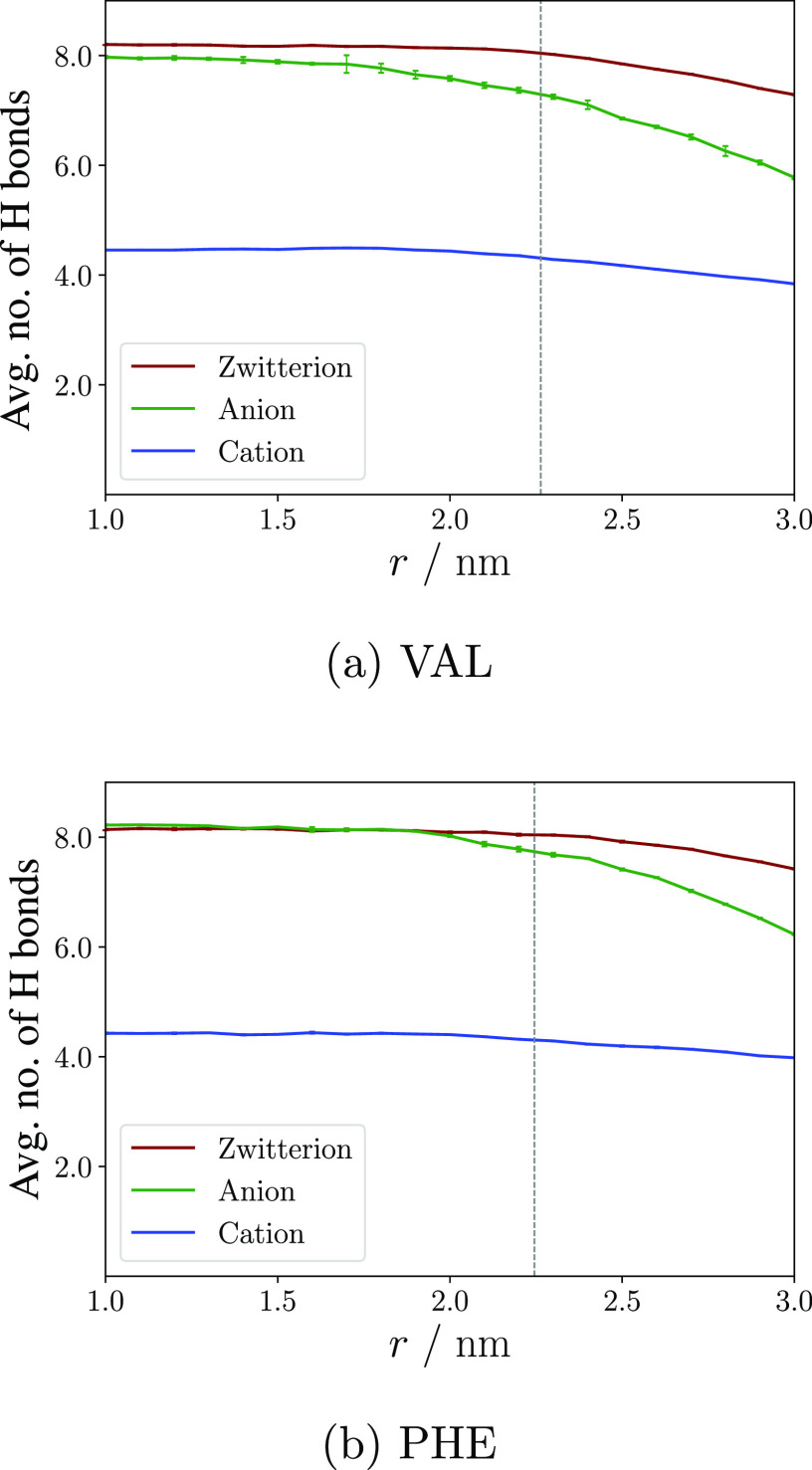
Average number of hydrogen bonds between VAL (a) and PHE ions (b) and water in the interphase region (cutoffs: distance *d* = 3.5 Å and angle θ = 30°). The reaction coordinate *r* is defined as the distance between the center of mass of the amino acid and the water slab. The dashed gray line indicates the Gibbs dividing surface.

Solvation of amino acids in water involves hydrogen bonding between water and the carboxyl and amine groups in the amino acid. Naturally, the prerequisites for hydrogen bonding depend strongly on charge state and protonation of the functional groups, which is highlighted by an analysis of the average number of hydrogen bonds between the amino acid and water. Figure 6 reveals that the anionic and zwitterionic states form an average of eight hydrogen bonds to neighboring water molecules in bulk, whereas the cationic state does not exceed an average of 4.5. This may at a first glance be attributed to enhanced hydrogen bonding properties of COO^–^, present in both the former, as compared to the protonated form, COOH, and the latter, which has been suggested by previous studies.^55^ The full picture is, however, more complex. The number of hydrogen bonds does clearly decrease for the anions as they lose their solvation shell when they approach the surface. The zwitterion, however, with the carboxyl group in the same protonation state as the anion, loses almost none of its hydrogen bonds at the surface. In fact, it is more similar to the cation in this regard, even though the latter is much less solvated. This explains the generally higher penalty for anion–water interaction in the left panel of Figure 5, as compared to the more moderate or even negative contributions for cations and zwitterions.

Looking at the individual electrostatic interactions of the carboxyl group and water versus the interaction of the amine group and water shows that the former gives positive contributions for all ions, i.e., the interaction of COO^–^/COOH with water, Δ*E*_c–w_, is always unfavorable at the surface (left panel of Figure 5). The interaction of the amine moiety Δ*E*_a–w_, however, gives significant stabilization in its protonated form (NH_3_^+^), seen for cation and zwitterion. The interaction of the carboxyl group hinders the surface location of the amino acid by a penalty of 9–16 kJ·mol^–1^ for the deprotonated species (anion, zwitterion) and around 2 kJ·mol^–1^ for the protonated form (cation). The interaction of the amine group instead shows a favorable contribution between −6 and −10 kJ·mol^–1^ for the protonated species (cation, zwitterion) but unfavorable around 11 kJ·mol^–1^ for the deprotonated form (anion). This explains the seemingly contradictory behavior of the zwitterion at the surface: it does not lose much of its solvation shell, but the unfavorable interactions of the deprotonated carboxyl group destabilize the molecule and make it less surface attracted.

Altogether, the protonated NH_3_^+^ group provides stable amino acid–water interactions at the surface, whereas carboxyl groups, especially in the COO^–^ form, interact unfavorably. Hence, the best combination is found within the cations (NH_3_^+^ and COOH) since they are able to use the favorable energy of the amine–water interaction without much limitation by the unfavorable carboxyl–water interaction. In addition, desolvation of VAL side chains at the surface is electrostatically favorable, whereas desolvation of PHE side chains is not, making VAL cations the most enthalpically favorable ion at the surface in this study (not taking the exceptional GLY zwitterions into account).

#### Amino Acid Intramolecular Interaction

The right panel of Figure 5 shows contributions from the intramolecular short-ranged Coulomb potential (including the specifically treated 1–4 interactions). Here, although most differences are small compared to both amino acid–water and water–water contributions, it is worth noting the intramolecular stabilization of PHE anion. This leads to the water–water interaction being advantageous, which explains the low enthalpy penalty seen for this ion in Figure 2.

Intramolecular interactions within PHE anion at the surface, and even more in vacuum, possess a hydrogen bonding character, i.e., the distance and orientation of interacting pairs is close to the geometric criteria for hydrogen bonding (cf. Figure S7d,f,h). It is probable that the strong intramolecular stabilization of PHE anions prevents hydrogen bonds to be formed between the amino acid and water molecules, which in turn, leads to more complete hydrogen bonding networks in water itself. This is supported by the very strong stabilization through water–water interaction in the PHE anion (cf. Figure 4). Advantageous intramolecular contribution for the VAL anion is seen as well, although less pronounced compared to PHE.

Strong intramolecular hydrogen bonding has previously been reported for zwitterionic amino acids.^56^ At a first glance, this feature was not apparent in the present study; when using the conventional geometric hydrogen bonding criteria (donor–acceptor distance <3.5 Å and hydrogen donor–acceptor (HDA) angle of 180 ± 30°), almost no intramolecular hydrogen bonds in zwitterions were detected. Even when considering all intramolecular atom pairs within 3.5 Å, the numbers were moderate (cf. Figure S5). Nevertheless, when considering the HDA angular distribution, it turned out that these angles were rather low for the zwitterion, indeed indicating strong and hydrogen bond-like interaction. Multiple peaks in the distribution also point to the fact that in the zwitterion all three HN atoms are participating in hydrogen bonds, whereas in the cation, a single preferred angle suggests that the interaction occurs mostly through the HO proton.

#### Nonspecific Interaction

Naturally, nonspecific interactions cannot be neglected in the investigation of the driving forces for surface propensity. Especially for the net charged ions, long-ranged electrostatic interactions will affect the cationic and anionic states not only at the surface, but also in bulk. This should best be reflected in the reciprocal Coulomb interactions modeled by the PME method. In the case of this study, the absolute reciprocal Coulomb energy was much smaller than the absolute short-range Coulomb contributions by a factor of almost 10^3^ and the difference between the former in bulk and at the surface was negligible. Nevertheless, water surfaces have been shown to be polarized due to the large dipole moment within molecules, resulting in an electrostatic potential gradient across the interface.^57^ The magnitude and sign of the potential in MD simulations is thus strongly dependent on the water model; moreover, the comparison with ab initio methods has shown that empirical force fields tend to misrepresent it altogether. In systems that include explicit treatment of electron density, smearing of said density into the vacuum is observed.^58^ This, together with local orientation of water molecules, renders the surface potential positive in all cases, whereas with the water model used here (SPC/E), negative surface potentials^57,59^ are observed, which was also the case in the present simulations. Some studies on hard-sphere models suggest that this will lead to an overestimation of the surface propensity, but it is not clear what effect it will have on real, soft-sphere systems.^59^ It was shown that the surface potential plays a major role in the surface behavior of small polarizable ions, where induced dipoles and charge delocalization complicate the picture.^60^ However, considering ion propensity at surfaces of SPC/E water, nonlocal effects seem to be outweighed by local electrostatics. The nonlocal electrostatic fluctuations are, moreover, nearly constant through the slab^58^ and therefore of less importance than the local.

The comparison of the subset simulated using GAFF/TIP3P with the results presented here showed that the conclusions hold true independent of the choice of the force field. The PMFs of anion and cation were similar to the ones obtained using OPLS-AA with SPC/E, but the zwitterion differed slightly. However, the surface propensity of zwitterions obtained with OPLS-AA could be validated by the literature^22^ (cf. Figure S9), and the construction of the zwitterion topology was not straightforward in GAFF, which might have led to the inaccuracy. Free energy decomposition of the GAFF simulations showed the same relationship between enthalpy and entropy as the corresponding simulations with OPLS-AA, thereby indicating force field independence.

## Conclusions

The surface propensities of three amino acids (GLY, VAL, and PHE) in three different charge states (anion, cation, and zwitterion) were calculated from MD simulations. While GLY was not likely to be found at water surfaces in any charge state, both VAL and PHE cations were found to be the most surface attracted, corresponding to an enhanced surface propensity of amino acids in water under acidic conditions. The complicated nature of surface propensity and the underlying mechanisms were highlighted, especially the balance act between enthalpy and entropy contributions and differences between the three amino acids as well as their charge states. VAL and PHE cations were the most attracted molecules due to a lower enthalpy penalty for VAL and entropy gain for PHE. A gain in the water–water enthalpy was found to be pronounced in all systems of VAL and PHE. Enthalpy contributions were further investigated in terms of electrostatic and dispersive interactions both between amino acids and water as well as within the amino acids. Special attention was drawn toward the differences between the three charge states, i.e., the presence of COO^–^, NH_3_^+^ as well as the uncharged COOH and NH_2_ and their abilities to form hydrogen bonds.

This, in turn, will help the understanding of the influence of pH on the surface propensity of free amino acids. Especially acidic aerosols will have more amino acids at their surface, strongly affecting both hygroscopicity and reactivity, indicating that pH is an important factor for cloud condensation dynamics. However, since temperatures in the atmosphere are likely lower than 300 K, temperature dependence is another interesting parameter that will have to be explored in future investigations. Based on the present results, we speculate that GLY zwitterions might be increasingly surface attracted at lower temperatures, while the propensity of both VAL and PHE will decrease, but the picture is complex and additional studies will have to be done on this matter.

## Supporting Information Available

Simulation details; description of the method used to determine the Gibbs dividing surface (includes Figure S1); additional figures to illustrate the free energy decomposition (Figure S2); specific interaction energies (Figures S3 and S4); hydrogen bonding (Figures S5 and S6); interaction angles (Figures S7 and S8); and a comparison with literature data (Figure S9) (PDF)

## Footnotes

a Since GLY shows no minima, the values were chosen at the average value of *r* where the minima of the other amino acids occurred.
