# Supplementary material for: Charge State Dependence of Amino Acid Propensity at Water Surface: Mechanisms Elucidated by Molecular Dynamics Simulations
Source: J Phys Chem A. 2021 May 27;125(22):4705–14. doi: 10.1021/acs.jpca.0c10963 (PMC8279654; doi:10.1021/acs.jpca.0c10963)
Supplement: Supplementary file 1 — jp0c10963_si_001.pdf [file jp0c10963_si_001.pdf]

Supporting Information

Charge State Dependence of Amino Acid Propensity at Water Surface - Mechanisms Elucidated by Molecular Dynamics Simulations

Radost Herboth,[†] Geethanjali Gopakumar,[‡] Carl Caleman,[‡,¶] and Malin Wohlert[†]

† Department of Materials Science and Engineering, Uppsala University, Box 35, 751 03, Uppsala, Sweden

‡ Department of Physics and Astronomy, Uppsala University, Box 516, 751 20 Uppsala, Sweden

¶ Center for Free-Electron Laser Science, DESY, Notkestraße 85, 226 07 Hamburg, Germany

Simulation details

Each system consisted of one amino acid in the water slab, that was first minimized using a steepest descent algorithm, then equilibrated 100 ps in an NPT simulation using the leap-frog algorithm with a timestep of 2 fs. Temperature and pressure were controlled by the Berendsen thermo- and barostat ($\tau_T = 0.1$ ps, $\tau_p = 2.0$ ps) at 300 K and 1 bar respectively. Evaporation of water molecules into the vacuum was controlled by a flat-bottom potential with a force constant of $k_{\mathrm{fb}} = 500\ \mathrm{kJ \cdot mol^{-1} \cdot nm^{-2}}$ and a cutoff of $r_{\mathrm{fb}} = 1.5$ Å; the amino acid was restrained to the value of the reaction coordinate by a harmonic potential, with a force constant of $1000\ \mathrm{kJ \cdot mol^{-1}\ nm^{-2}}$ in every direction. All bonds were constrained with the LINCS algorithm in a 4th order expansion (1 iteration to correct for rotational

lengthening). Neighborlist grid searching was used for non-bonded interactions with a cutoff of 1.4 nm, the lists were updated every 5 steps or every 10 fs. Long-range electrostatics were described by particle-mesh Ewald (PME)[1] with the same cutoff, a cubic interpolation and grid spacing of 0.16 nm. A long-range dispersion correction was furthermore applied to energy and pressure.

Pulling in the steered MD simulation proceeded in z-direction at a rate of 0.01 nm $\cdot$ ps^{-1} from the center ($r = 0$ nm) into the vacuum ($r = 5$ nm), with a force constant of 10000 kJ $\cdot$ mol^{-1} $\cdot$ nm^{-2}. The simulation was thus run for a total of 500 ps. Stochastic dynamics was used with a timestep of 2 fs. Temperature coupling was then implicit ($T_0 = 300$ K) and pressure coupling turned off. While the pulling simulation proceeded from $r = 0$ nm to $r = 5$ nm, only configurations between 1 and 5 nm were used in umbrella sampling. The umbrella simulations were run in an NVT ensemble with stochastic dynamics as the integrator ($\Delta t = 2$ fs). Temperature coupling was implicit and pressure coupling turned off, the reference temperature is 300 K (τ_T is 0.1 ps for equilibration and 1.0 ps for production). All bonds were constrained with LINCS, with the same parameters as in the first equilibration. Non-bonded interactions were controlled by neighborlist grid searching and the PME method. The cutoff for all interactions was 1.4 nm ; lists were updated every 5 steps (or 10 fs) and every 20 steps (or 40 fs) for equilibration and production respectively and PME was performed using a cubic interpolation and grid spacing of 0.16 nm (equilibration) or 0.12 nm (production). As in the first equilibration, a long-range dispersion correction was applied to energy and pressure. The amino acid was position restrained as before in the equilibration of the umbrella simulations, while in the production run the harmonic umbrella potential with a force constant of 1000 kJ $\cdot$ mol^{-1} $\cdot$ nm^{-2} was applied.

PMF error estimates were obtained from Bayesian bootstrapping with 100 generated datasets. For faster convergence, only the steps every 10 ps were used in WHAM for the GLY ions.

Further methods

The Gibbs dividing surface was determined by calculating the density of the water slab along the the z direction of the simulation box. Then, the interphase region was divided into two parts and the position of this 'divider' was optimized so that the area between the density curve and the vertical divide would be identical on either side (cf. Figure S1). This was done by fitting the density curve in the interphase region using a 10th order polynomial (dashed gray line), integrating the obtained function on either side of the divide, subtracting the integral on the left from the total area between bulk density and divide (see light gray lines) and comparing the resulting areas (dark blue shades). If the difference between the two areas was sufficiently small, i.e. they were near-identical, the optimization was stopped and the position of the divider was recorded as the Gibbs dividing surface.

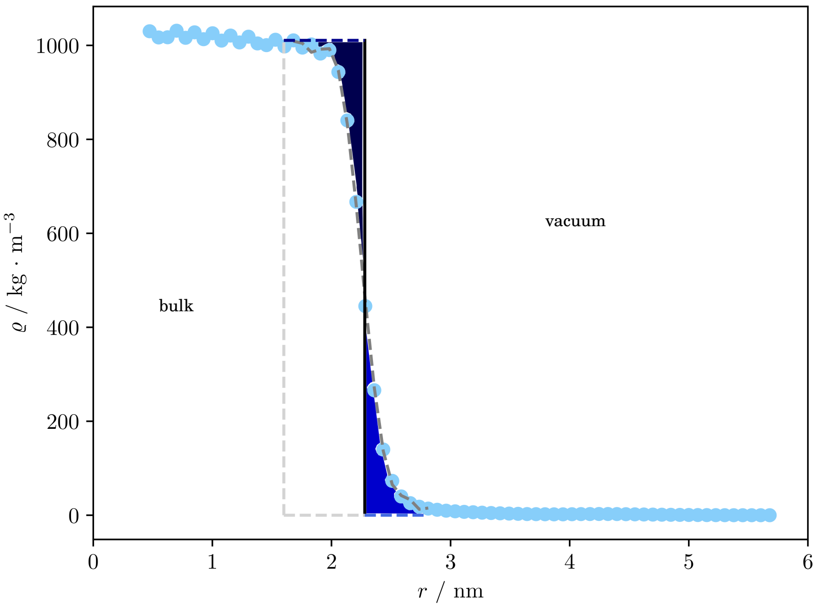

Figure S1: Light blue dots: Water density as a function of the reaction coordinate r, which is defined as the distance between the center of mass of the amino acid and the center of mass of the water slab. The dashed dark gray line marks the 10th order polynomial fitted to the density curve in the interphase region, while the dashed light gray line marks an auxiliary line used in the calculation of the Gibbs dividing surface (see text). The optimized position of the Gibbs dividing surface is marked by a solid black line, with the areas used in optimization as dark blue shades left and right.

Additional Figures

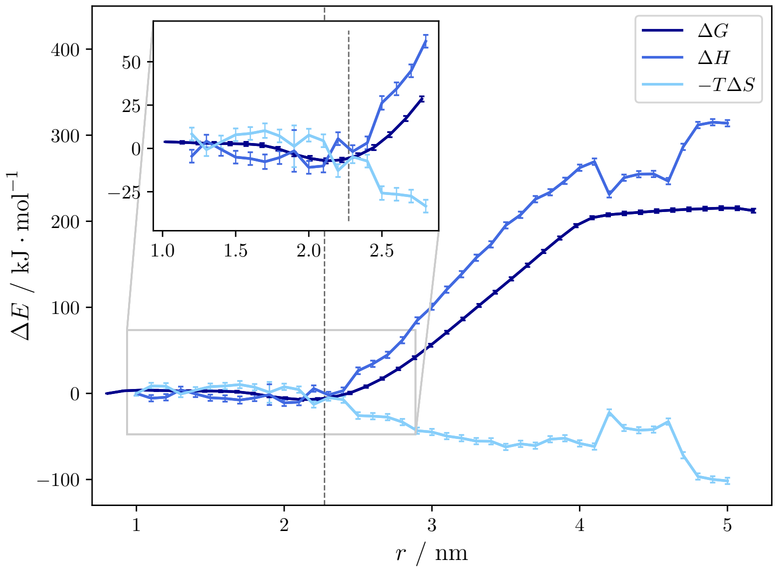

(a) VAL

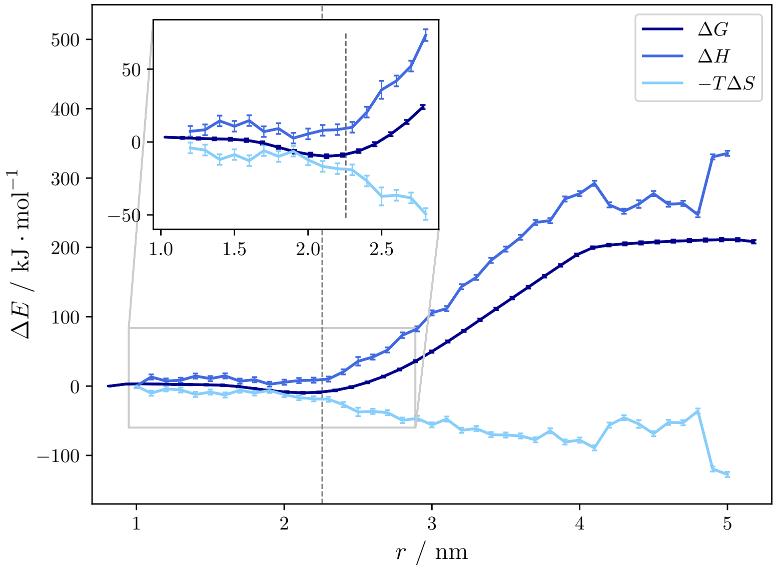

(b) PHE

Figure S2: Energy decomposition of VAL (a) and PHE (b) **cations** at the interface according to Equation 1 over the reaction coordinate r (distance between the center of mass of amino acid and water slab). The term ΔG is the relative free energy (point of reference: $r = 1.0$ nm) obtained from umbrella sampling and also the sum of the other contributions; ΔH is the relative enthalpy contribution and $-T\Delta S$ is the relative entropy contribution with reference to the same point.

Specific interactions amino acid-water

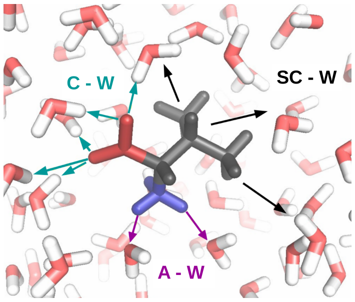

C-W: carboxyl-water interaction
A-W: amine-water interaction
SC-W: sidechain-water interaction

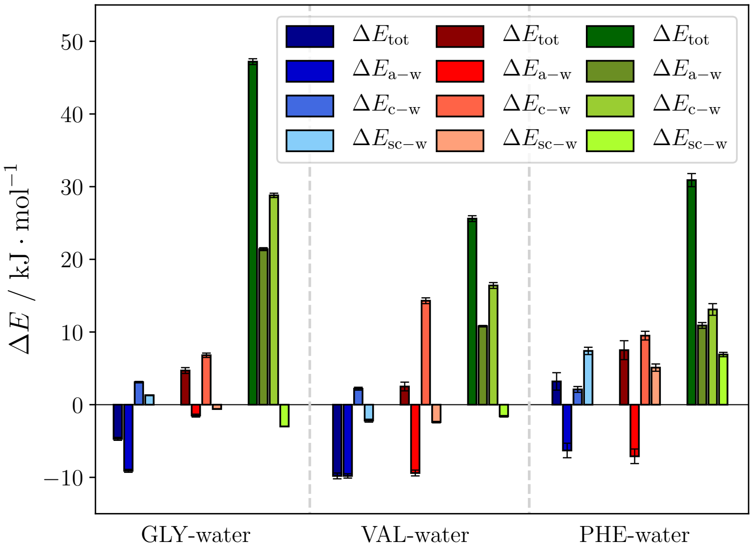

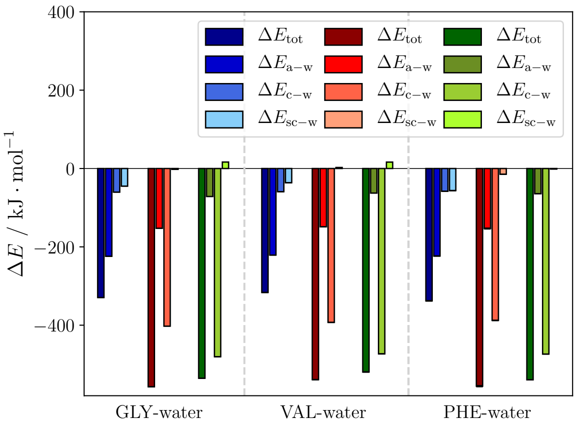

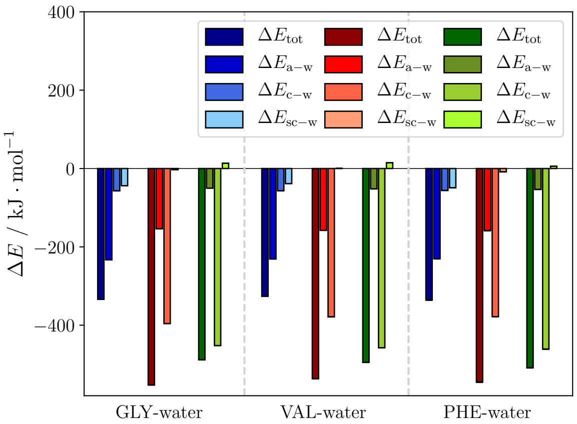

Figure S3: **Top left**: Pictorial view of the contributions to amino acid-water interaction. The carboxyl group is present as the protonated form COOH in the cation and deprotonated as COO^- in anion and zwitterion, whereas the amine group occurs as NH_2 in the anion and in the protonated form NH_3^+ in cation and zwitterion (pictured: VAL zwitterion). **Top right**: Contributions from each group in the illustration to the left to the amino acid-water enthalpy differnece between surface and bulk. The total interaction energy is given by the short-ranged Coulomb interaction, all energies are given as the difference between PMF minimum and the first point of the PMF ($r = 1.0$ nm). Color coding: Each color group denotes one charge state, blue for the cationic state, red for the zwitterionic and green for the anionic state. **Bottom**: Absolute short-ranged Coulomb interaction energy in bulk (**left panel**) and at the surface (**right panel**) for amino acid-water interactions (tot: total interaction energy, other interactions and color coding as above).

Specific intramolecular interactions in the amino acid

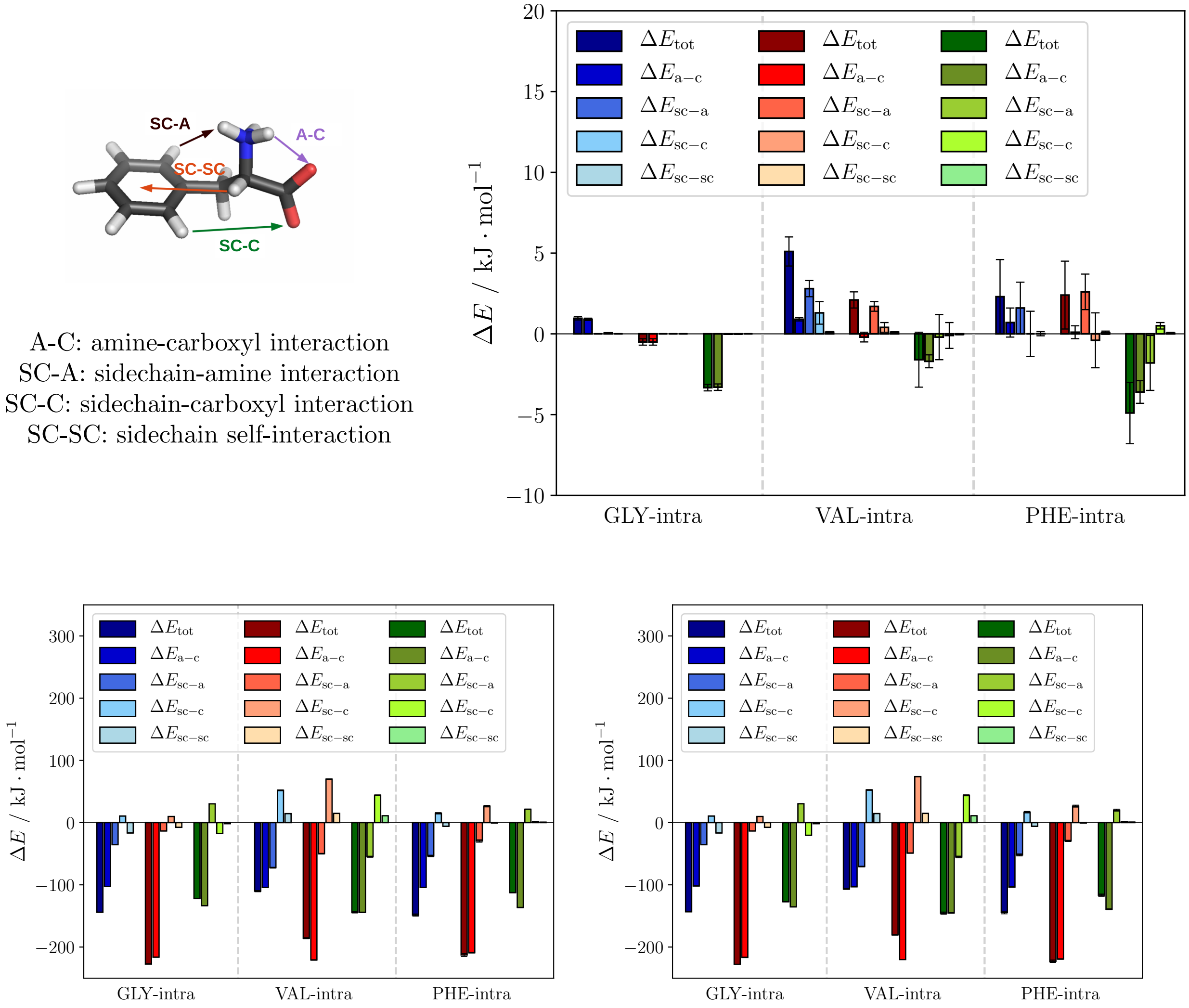

Figure S4: **Top left**: Pictorial view of the contributions to the intramolecular interaction within the amino acid. Groups are defined the same way as in Figure S3 (pictured: PHE zwitterion). **Top right**: Contributions from each group in the illustration to the left to intramolecular enthalpy difference between surface and bulk. The total interaction energy here is the sum of short-ranged and 1-4 intramolecular Coulomb interaction energies, all energies are given as the difference between PMF minimum and the first point of the PMF ($r = 1.0$ nm). Color coding: Each color group denotes one charge state, blue for the cationic state, red for the zwitterionic and green for the anionic state. **Bottom**: Absolute sum of short-ranged and 1-4 intramolecular Coulomb interactions energy in bulk (**left panel**) and at the surface (**right panel**) for all amino acids (tot: total interaction energy, other interactions and color coding as above).

Specific interaction amino acid (hydrogen bonds)

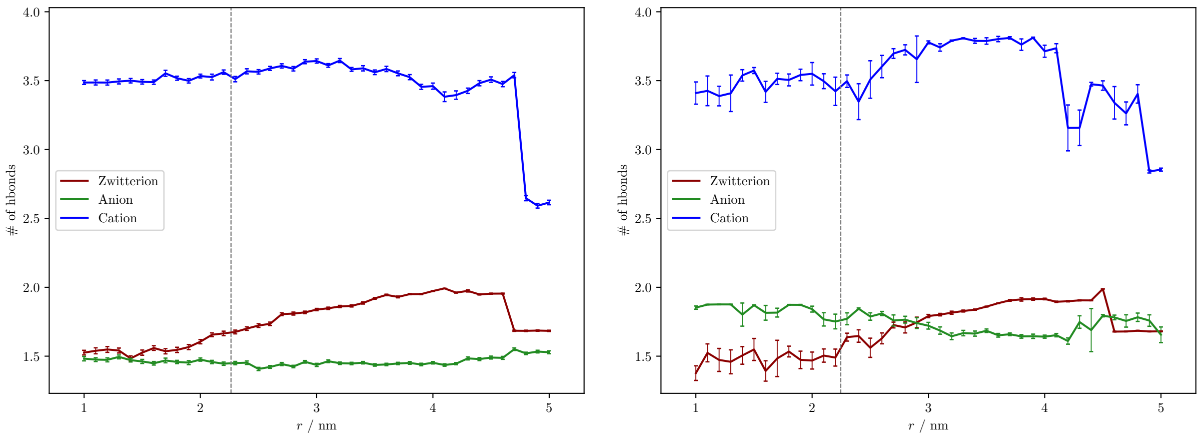

(a) VAL (b) PHE

Figure S5: Average number of intramolecular interaction pairs in VAL (a) and PHE ions (b) over the reaction coordinate (cutoffs: distance $d = 3.5$ Å, angle $\theta = 180°$). The reaction coordinate r is defined as the distance between the center of mass of amino acid and water slab. The dashed grey line indicates the Gibbs dividing surface.

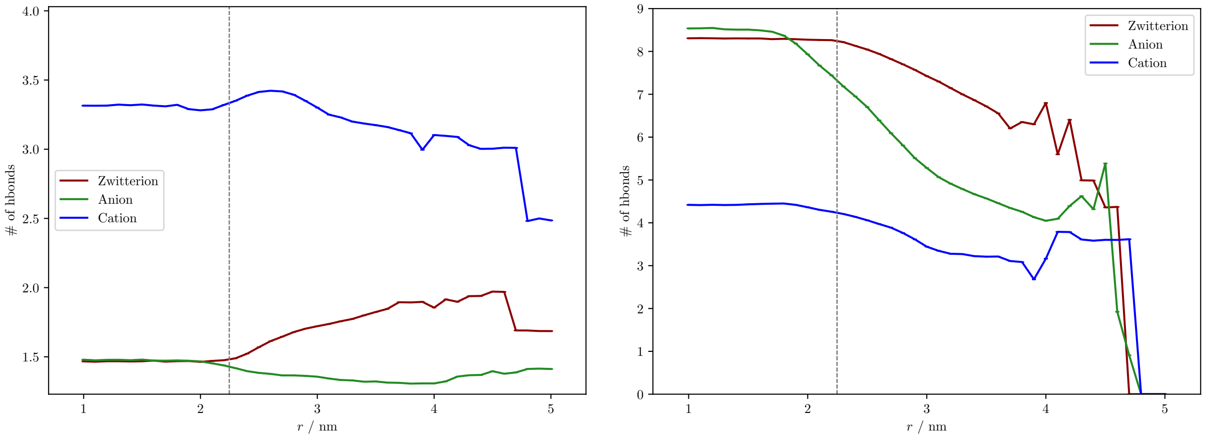

(a) GLY intra (b) GLY-water

Figure S6: (a) Average number of intramolecular interaction pairs in GLY ions over the reaction coordinate r (cutoffs: distance $d = 3.5$ Å, angle $\theta = 180°$). The dashed grey line indicates the Gibbs dividing surface. (b) Average number of hydrogen bonds between GLY and water over the reaction coordinate (cutoffs: distance $d = 3.5$ Å, angle $\theta = 30°$). The reaction coordinate r is defined as the distance between the center of mass of amino acid and water slab. The dashed grey line indicates the Gibbs dividing surface.

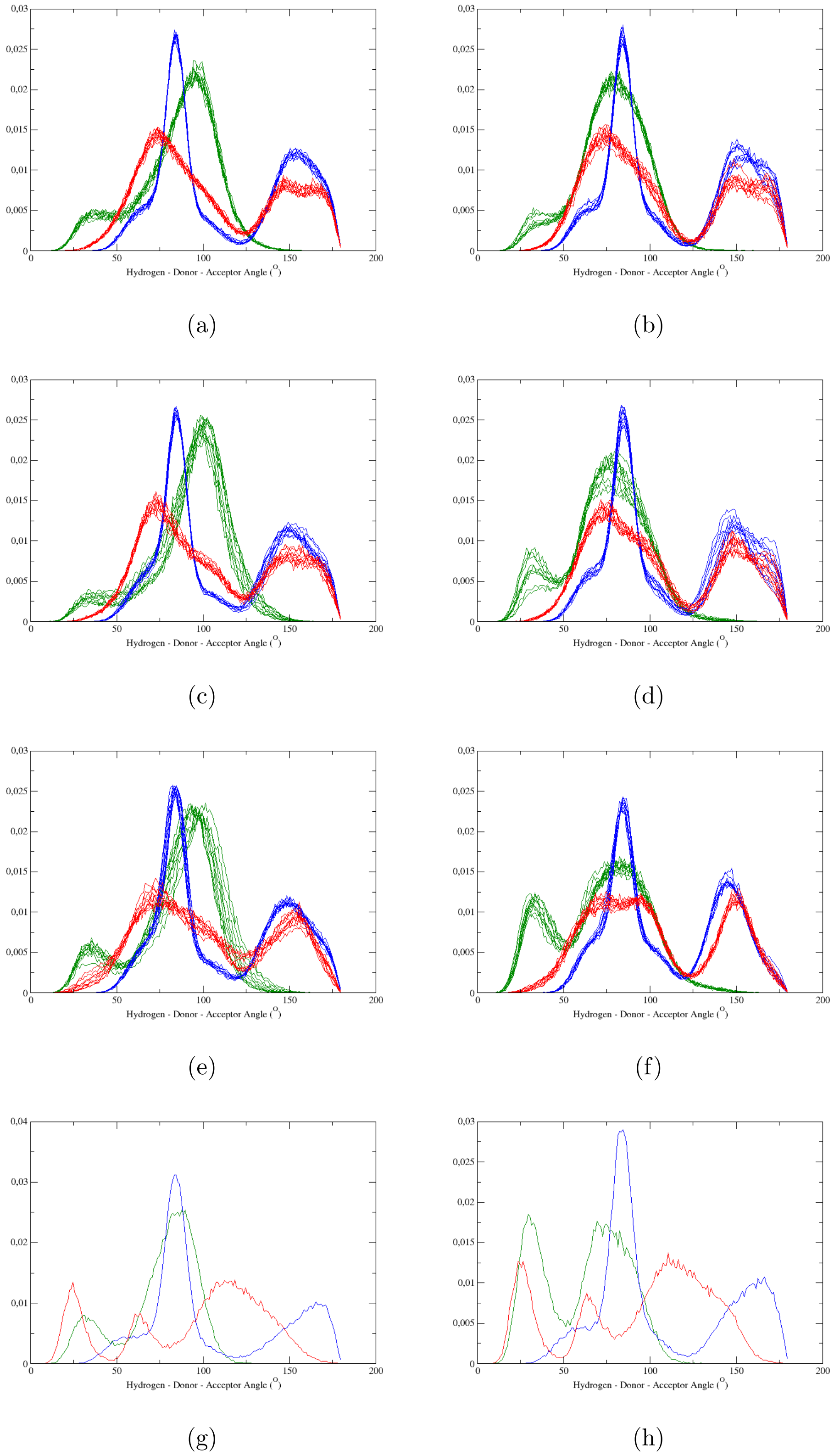

Figure S7: Intramolecular interaction angle distributions for VAL (left panels) and PHE (right panels): a, b bulk (1.0 – 1.9 nm); c, d surface (2.0 – 2.9 nm); e, f near-surface vacuum (3.0 – 3.9 nm); g, h vacuum (5.0 nm). Color coding as before: green anion, blue cation, red zwitterion.

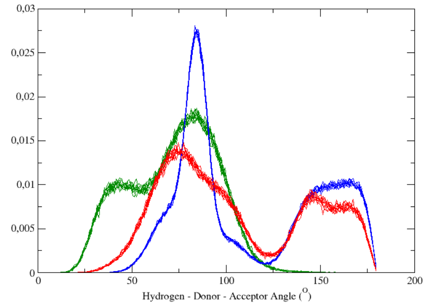

(a)

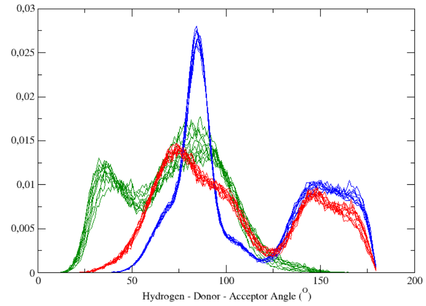

(b)

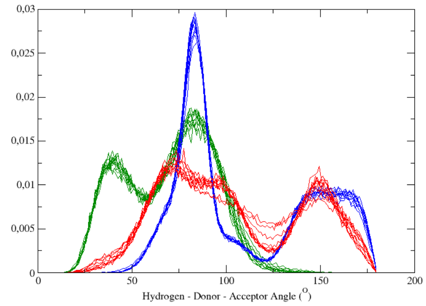

(c)

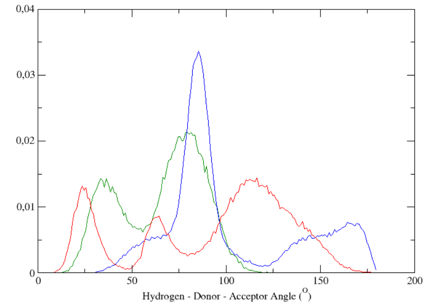

(d)

Figure S8: Intramolecular interaction angle distributions for GLY: a bulk (1.0 – 1.9 nm); b surface (2.0 – 2.9 nm); c near-surface vacuum (3.0 – 3.9 nm); d vacuum (5.0 nm). Color coding as before: green anion, blue cation, red zwitterion.

Comparison with literature

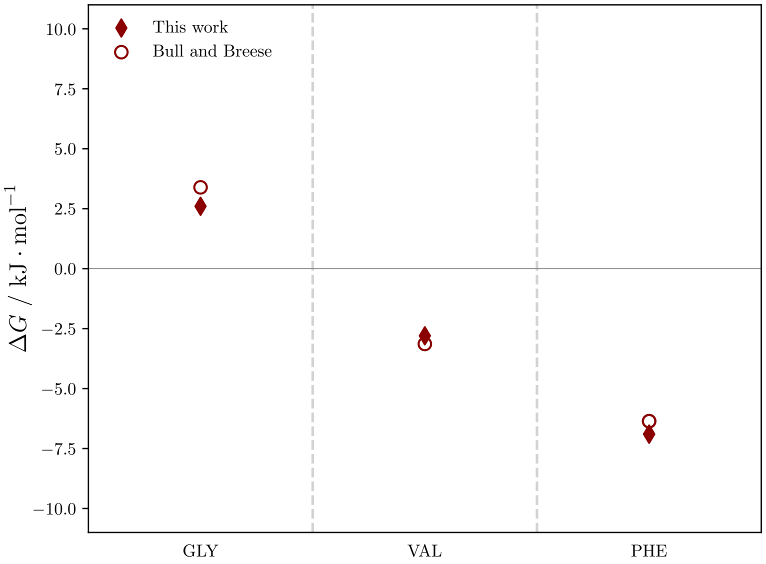

Figure S9: Comparison of the values for the free energy at the PMF minimum (filled diamonds) and literature data for the free energy of transfer to the surface obtained from surface tension measurements by Bull and Breese[2] (circles) for the **zwitterionic** states of GLY, VAL and PHE.

References

(1) Darden, T.; York, D.; Pedersen, L. Particle mesh Ewald: An $N \cdot \log(N)$ method for Ewald sums in large systems. *The Journal of Chemical Physics* **1993**, *98*, 10089–10092.

(2) Bull, H. B.; Breese, K. Surface tension of amino acid solutions: A hydrophobicity scale of the amino acid residues. *Archives of Biochemistry and Biophysics* **1974**, *161*, 665–670.
